# Correlations between 6-minute walk test, chair-rise test, and lower extremity functional scale among patients with hypophosphatasia

**DOI:** 10.1016/j.bonr.2025.101853

**Published:** 2025-05-29

**Authors:** Lothar Seefried, Franca Genest

**Affiliations:** University of Würzburg, Würzburg, Germany

**Keywords:** Hypophosphatasia, Rare disease, 6-minute walk test, Chair-rise test, Lower extremity functional scale, Clinical assessment

## Abstract

**Purpose:**

Hypophosphatasia (HPP) is a rare disease characterized by skeletal and nonskeletal manifestations that can increase patient disability. The 6-Minute Walk Test (6MWT) is frequently used to assess mobility in patients with HPP, although the test is laborious to conduct in clinical practice. The purpose of the current study was to determine correlations between time to complete the 6MWT, time to complete the Chair-Rise Test (CRT), and scores on the Lower Extremity Functional Scale (LEFS) in adults with HPP.

**Methods:**

Pearson correlations between time to complete outcomes on the 6MWT and CRT, time to complete the 6MWT and scores on the LEFS, and time to complete the CRT and scores on the LEFS were calculated using de-identified data from adults with HPP who had first onset of symptoms in childhood. All patients were enrolled in the previously conducted, observational EmPATHY study.

**Results:**

Pearson correlation analyses showed inverse correlations between 6MWT and CRT outcomes (*r* = −0.584) and between CRT and LEFS outcomes (*r* = −0.596) and a direct correlation between 6MWT and LEFS outcomes (*r* = 0.808).

**Conclusions:**

Time to complete the 6MWT was correlated with time to complete the CRT and scores on the LEFS in adults with HPP. CRT and LEFS may be suitable, expeditious options to amend or substitute 6MWT when assessing functional status in patients with HPP.

## Highlights

• The 6-Minute Walk Test (6MWT) is time-consuming to perform in clinical practice
• Chair-Rise Test (CRT) and Lower Extremity Functional Scale (LEFS) may be faster
• Outcomes of the 6MWT correlated with CRT and LEFS outcomes in patients with HPP
• Clinicians may consider CRT or LEFS as alternative evaluations for HPP

## 1 Introduction

Hypophosphatasia (HPP) is a rare metabolic disease caused by deficient tissue non-specific alkaline phosphatase (ALP) activity (Seefried et al., 2020). HPP is characterized by a wide variety of clinical signs and symptoms, including both skeletal and nonskeletal manifestations (Conti et al., 2017). Patients with HPP may experience compromised bone mineralization, muscle weakness, and chronic pain, which lead to high levels of disability and poor quality of life (Seefried et al., 2020; Kishnani et al., 2017). High proportions of patients report employing adaptive strategies for disability, including use of assistive devices (e.g., canes, crutches, walkers) and home modifications (e.g., handrails, bath/shower modifications) (Seefried et al., 2020; Weber et al., 2016).

One of the validated functional tests used to assess patients with HPP is the 6-Minute Walk Test (6MWT) (Phillips et al., 2019). This test measures the distance a patient can walk on a hard, flat surface over a period of 6 min, and is thus a measure of walking ability and fatiguability (Phillips et al., 2019; American Thoracic Society, 2002). Despite its utility, the 6MWT test has some limitations in clinical practice. For example, a treadmill should not be used for the test (American Thoracic Society, 2002). Instead, healthcare providers should administer the test in a 100-ft hallway that is marked every 3 m and is not frequently traveled. The test also takes a relatively long time to perform (American Thoracic Society, 2002).

The Chair-Rise Test (CRT) and Lower Extremity Functional Scale (LEFS) are potential alternatives to the 6MWT. The CRT is part of the Short Physical Performance Battery and measures how long it takes for a patient to stand up from a chair five times, as quickly as possible, without hand support (Zhang et al., 2013; Guralnik et al., 1994). The LEFS is a survey consisting of 20 questions that indicate functioning of the lower extremities (Binkley et al., 1999). The scale can be completed by the patient and scored by the practitioner in <3 min (Binkley et al., 1999). As such, both the CRT and LEFS functional assessments are fast, easy, and practical ways to assess the strength of the proximal thigh muscles, endurance, and lower extremity functionality.

The objective of the current analysis was to determine whether there is a correlation between distance walked in the 6MWT and time to complete the CRT or LEFS score among adults with HPP. While a correlation between 6MWT and CRT has been previously demonstrated in healthy patients and those with COPD (Höglund et al., 2022; Meriem et al., 2015; Gurses et al., 2018), this has not been evaluated in patients with HPP. This analysis used data from the previously published Evaluate and Monitor Physical Performance of Adults Treated With Asfotase Alfa for Hypophosphatasia (EmPATHY) study (Genest et al., 2020; Seefried et al., 2021; Seefried et al., 2023).

## 2 Methods

### 2.1 Patients

Data for this analysis (6MWT, CRT, and LEFS) were collected from 22 patients (17 female, 5 male) enrolled in the EmPATHY study (Genest et al., 2020; Seefried et al., 2021; Seefried et al., 2023). EmPATHY was an observational study of patients with HPP who received treatment with asfotase alfa, a tissue-nonspecific ALP enzyme replacement therapy, during routine clinical care at the University of Würzburg. Patients included in the EmPATHY study were adults ≥18 years old who first presented with signs and symptoms of HPP in childhood. All patients provided informed consent before enrollment in the EmPATHY study. All procedures were performed in compliance with relevant laws and institutional guidelines and were approved by the ethics committee of the University of Würzburg, Germany (No. 9/18).

### 2.2 Correlation analysis

Correlation analyses were performed using 6MWT, CRT, and LEFS data obtained on the same day from individual patients at different visits during the EmPATHY study, including at baseline (prior to starting asfotase alfa treatment) and at months 3, 6, and every 6 months of treatment thereafter. Pearson correlation analyses were performed for all assessments. All patient data were deidentified for analysis. All statistical analyses were performed with SAS.

## 3 Results

**Fig. 1:**
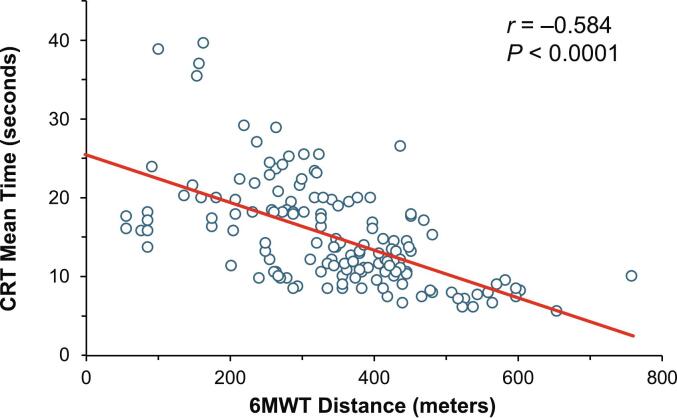
Pearson correlation of 6MWT distance walked and time to complete CRT.

Among all analyzed patient data, 175 assessments (approximately 8 per patient) were available. Paired data on CRT vs. 6MWT, CRT vs. LEFS and 6MWT vs. LEFS were available for 149, 147, and 172 assessments, respectively. The overall time to complete the CRT ranged from 5.3 to 39.4 s and the overall range of 6MWT distances walked was 0 to 760 m. There was a statistically significant inverse relationship between distance walked on the 6MWT and time to complete the CRT (*r* = −0.584, *P* < 0.0001; Fig. 1).

**Fig. 2:**
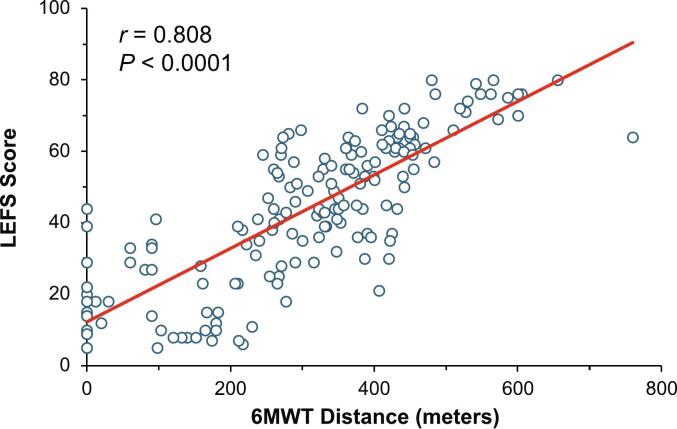
Pearson correlation of 6MWT distance walked and LEFS score.

**Fig. 3:**
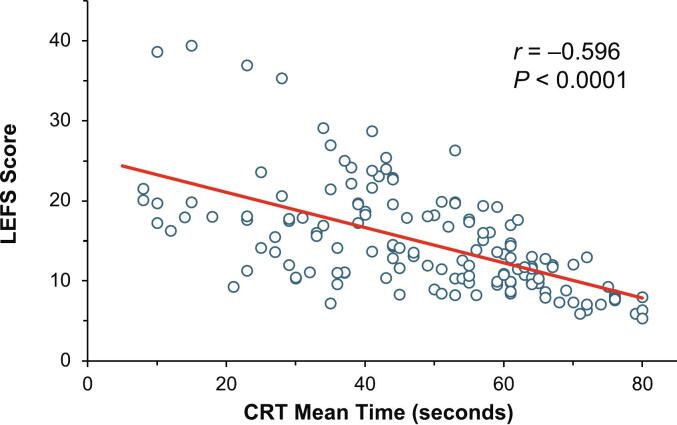
Pearson correlation of time to complete CRT and LEFS score.

The overall score on the LEFS ranged from 5 to 80 among all patients. There was a statistically significant direct relationship between distance walked on the 6MWT and LEFS score in patients with HPP (*r* = 0.808, *P* < 0.0001; Fig. 2). A Pearson correlation analysis also showed a statistically significant inverse relationship between time to complete the CRT and LEFS score (*r* = −0.596, *P* < 0.0001; Fig. 3).

## 4 Discussion

The results of this analysis show that distance walked on the 6MWT is correlated with time to complete the CRT and LEFS score among adults with HPP. These data indicate that the CRT and LEFS may be suitable alternatives to the 6MWT for assessing functionality in adults with HPP. CRT and LEFS are faster and easier to administer than the 6MWT (Zhang et al., 2013; Guralnik et al., 1994; Binkley et al., 1999), making them attractive alternatives in clinical practice.

Other analyses have shown correlations between distance walked on the 6MWT and clinical outcomes. A significant inverse correlation was found between 6MWT distance walked and CHAQ-DI scores in children with HPP (Phillips et al., 2019). Significant direct correlations were found between 6MWT distance walked and PODCI scores in children with HPP, and between 6MWT distance walked and LEFS score in adolescents and adults with HPP (Phillips et al., 2019). Among patients with HPP who were treated with asfotase alfa, outcomes on the 6MWT, CRT, and LEFS assessments improved over up to 2 years of treatment (Genest et al., 2020; Seefried et al., 2023).

Among healthy adults, mean time to complete the CRT was 8.2 s (range 4.9 to 12.7 s) for those aged <60 years of age and 13.4 s (range 7.5 to 19.6 s) for those >60 years of age (Whitney et al., 2005). In the current study, the time to complete the CRT ranged from 5.3 to 39.4 s, suggesting that the time to complete the CRT is longer for many patients with HPP than in a healthy adult population. The mean LEFS score in a healthy population analysis was 69 (range 4.5 to 80) (Dingemans et al., 2017), which was similar to the range among patients with HPP in the current study (range 5 to 80).

This analysis has some limitations. Mean time to complete the CRT and mean LEFS score were not calculated in the current study, limiting comparisons that can be drawn to healthy population estimates. The EmPATHY trial enrolled a relatively small cohort of patients, although correlation data are available at multiple timepoints for each patient throughout the 2 years of study, so some caution should be used when extrapolating our findings. The EmPATHY study also only enrolled adults aged ≥18 years with HPP, so further research is required to determine if a correlation exists between distance walked in the 6MWT and either the CRT or LEFS score in children with HPP.

## 5 Conclusion

The correlations between 6MWT, CRT, and LEFS are clinically useful findings in adults with HPP. Since the CRT can typically be completed in under one minute and only requires a chair, this test may be a useful alternative to the 6MWT. Similarly, the LEFS survey can be completed and scored in under 3 min, with no requirements for equipment. Each of these assessments can be suitable alternatives that correlate with distance walked in the 6MWT and may be considered by clinicians who evaluate patients with HPP.

## CRediT authorship contribution statement

**Lothar Seefried:** Conceptualization, Data curation, Formal analysis, Investigation, Methodology, Supervision, Writing – review & editing. **Franca Genest:** Data curation, Formal analysis, Investigation, Methodology, Writing – review & editing.

## Funding

10.13039/100006396Alexion, AstraZeneca Rare Disease supported the research and medical writing as an externally sponsored research project. Medical writing and editorial assistance was provided by Peloton Advantage, LLC, an OPEN Health company.

## Declaration of competing interest

**LS** is a clinical study investigator and has received consultancy fees and institutional research funding and/or grant support from Alexion, AstraZeneca Rare Disease; Amgen; AM-Pharma; BioMarin; Chiesi; Haleon/GSK; Inozyme; Ipsen; Kyowa Kirin; MediPharma; Novartis; STADApharm; Theramex; UCB; and Ultragenyx.

**FG** received speaker honoraria from Alexion.

## Data Availability

Qualified academic investigators may request deidentified data and supporting documents pertaining to this study from the corresponding author (LS).

## References

[bb0005] American Thoracic Society (2002). ATS statement: guidelines for the six-minute walk test. Am. J. Respir. Crit. Care Med..

[bb0010] Binkley J.M., Stratford P.W., Lott S.A., Riddle D.L. (1999). The lower extremity functional scale (LEFS): scale development, measurement properties, and clinical application. North American Orthopaedic rehabilitation research network. Phys. Ther..

[bb0015] Conti F., Ciullini L., Pugliese G. (2017). Hypophosphatasia: clinical manifestation and burden of disease in adult patients. Clin. Cases Miner. Bone Metab..

[bb0020] Dingemans S.A., Kleipool S.C., Mulders M.A.M., Winkelhagen J., Schep N.W.L., Goslings J.C., Schepers T. (2017). Normative data for the lower extremity functional scale (LEFS). Acta Orthop..

[bb0025] Genest F., Rak D., Petryk A., Seefried L. (2020). Physical function and health-related quality of life in adults treated with asfotase alfa for pediatric-onset hypophosphatasia. JBMR Plus.

[bb0030] Guralnik J.M., Simonsick E.M., Ferrucci L., Glynn R.J., Berkman L.F., Blazer D.G., Scherr P.A., Wallace R.B. (1994). A short physical performance battery assessing lower extremity function: association with self-reported disability and prediction of mortality and nursing home admission. J. Gerontol..

[bb0035] Gurses H.N., Zeren M., Denizoglu Kulli H., Durgut E. (2018). The relationship of sit-to-stand tests with 6-minute walk test in healthy young adults. Medicine (Baltimore).

[bb0040] Höglund J., Boström C., Sundh J. (2022). Six-minute walking test and 30 seconds chair-stand-test as predictors of mortality in COPD - a cohort study. Int. J. Chron. Obstruct. Pulmon. Dis..

[bb0045] Kishnani P.S., Rush E.T., Arundel P., Bishop N., Dahir K., Fraser W., Harmatz P., Linglart A., Munns C.F., Nunes M.E., Saal H.M., Seefried L., Ozono K. (2017). Monitoring guidance for patients with hypophosphatasia treated with asfotase alfa. Mol. Genet. Metab..

[bb0050] Meriem M., Cherif J., Toujani S., Ouahchi Y., Hmida A.B., Beji M. (2015). Sit-to-stand test and 6-min walking test correlation in patients with chronic obstructive pulmonary disease. Ann. Thorac. Med..

[bb0055] Phillips D., Tomazos I.C., Moseley S., L'Italien G., Gomes Da Silva H., Lerma Lara S. (2019). Reliability and validity of the 6-minute walk test in hypophosphatasia. JBMR Plus.

[bb0060] Seefried L., Dahir K., Petryk A., Högler W., Linglart A., Martos-Moreno G.Á., Ozono K., Fang S., Rockman-Greenberg C., Kishnani P.S. (2020). Burden of illness in adults with hypophosphatasia: data from the global Hypophosphatasia patient registry. J. Bone Miner. Res..

[bb0065] Seefried L., Rak D., Petryk A., Genest F. (2021). Bone turnover and mineral metabolism in adult patients with hypophosphatasia treated with asfotase alfa. Osteoporos. Int..

[bb0070] Seefried L., Genest F., Petryk A., Veith M. (2023). Effects of asfotase alfa in adults with pediatric-onset hypophosphatasia over 24 months of treatment. Bone.

[bb0075] Weber T.J., Sawyer E.K., Moseley S., Odrljin T., Kishnani P.S. (2016). Burden of disease in adult patients with hypophosphatasia: results from two patient-reported surveys. Metabolism.

[bb0080] Whitney S.L., Wrisley D.M., Marchetti G.F., Gee M.A., Redfern M.S., Furman J.M. (2005). Clinical measurement of sit-to-stand performance in people with balance disorders: validity of data for the five-times-sit-to-stand test. Phys. Ther..

[bb0085] Zhang F., Ferrucci L., Culham E., Metter E.J., Guralnik J., Deshpande N. (2013). Performance on five times sit-to-stand task as a predictor of subsequent falls and disability in older persons. J. Aging Health.

